# Donor Specific Antibodies in Extracorporeal Membrane Oxygenation-Bridged Lung Transplant Recipients

**DOI:** 10.1016/j.atssr.2024.06.021

**Published:** 2024-07-04

**Authors:** Lydia E. Federico, Joshua M. Diamond, Malek Kamoun, Maria M. Crespo, Christian A. Bermudez, Andrew M. Courtwright

**Affiliations:** 1 Perelman School of Medicine, University of Pennsylvania, Philadelphia, Pennsylvania; 2 Department of Pulmonary and Critical Care Medicine, University of Pennsylvania, Philadelphia, Pennsylvania; 3 Department of Pathology and Laboratory Medicine, University of Pennsylvania, Philadelphia, Pennsylvania; 4 Department of Cardiovascular Surgery, University of Pennsylvania, Philadelphia, Pennsylvania

## Abstract

**Background:**

Extracorporeal membrane oxygenation (ECMO) is increasingly used as a bridge to lung transplantation. Although other mechanical circulatory support devices have been associated with anti-human leukocyte antigen antibody formation, including de novo donor-specific antibodies (dnDSA), it is unknown whether ECMO is a sensitizing exposure.

**Methods:**

This was a single-center retrospective cohort study of lung transplant recipients. We compared dnDSA development in ECMO-bridged and non-ECMO exposed recipients. We also assessed differences in chronic lung allograft dysfunction-free survival between ECMO-bridged recipients with and without dnDSA, and between those who developed dnDSA with and without ECMO bridge.

**Results:**

Among 299 transplant recipients, 48 were ECMO-bridged and 251 were non-ECMO exposed. dnDSA developed in 33.3% of ECMO-bridged and 21.5% of non-ECMO exposed recipients. ECMO was associated with dnDSA development in bivariate (hazard ratio [HR], 2.08, 95% CI, 1.19-3.64, *P* = .01) but not multivariate analysis after adjusting for known confounders (HR, 1.10, 95% CI, 0.50-2.42, *P* = .82). Higher posttransplant transfusion volume was independently associated with dnDSA development (HR, 4.68, 95% CI, 2.25-9.72, *P* < .001). ECMO-bridged recipients with dnDSA did not have worse adjusted chronic lung allograft dysfunction-free survival than those without dnDSA (HR, 0.35, 95% CI, 0.07-1.87, *P* = .22) or compared to non-ECMO exposed recipients with dnDSA (HR, 0.40, 95% CI, 0.08-2.00, *P* = .27).

**Conclusions:**

A more restrictive posttransplant transfusion threshold among ECMO-bridged lung transplant recipients may reduce the risk of dnDSA. Surveillance for dnDSA, at least among ECMO-bridged recipients, may only be necessary in the presence of allograft dysfunction.

**In Short**

▪ Posttransplant transfusions but not extracorporeal membrane oxygenation (ECMO) bridge were associated with de novo donor-specific antibody formation in lung transplant recipients.
▪ De novo donor-specific antibodies among ECMO-bridged lung transplant recipients did not impact chronic lung allograft dysfunction-free survival.

Extracorporeal membrane oxygenation (ECMO) is increasingly used as a bridge to lung transplantation. However, mechanical circulatory devices expose patients to biomaterial, which may elicit host immunologic responses. For example, ventricular assist devices are associated with anti-human leukocyte antigen (HLA) antibody development in heart transplant candidates, which appears to be independent of blood transfusion, possibly due to host interactions with device biomaterial.^1^

There is limited research investigating whether ECMO is also independently associated with sensitization. We recently reported that ECMO-associated sensitization while awaiting lung transplant is primarily attributable to increased likelihood of receiving transfusions.^2^ Given the short duration of ECMO, however, ECMO-induced B-cell hyperreactivity may only be observed posttransplant. Alternatively, ECMO-related B-cell priming may increase risk of de novo donor-specific HLA antibody (dnDSA) formation. For example, Ius and associates^3^ identified pretransplant ECMO as a risk factor for dnDSAs.^3^ Only 10% of their cohort, however, had ECMO exposure and they did not account for transfusions, limiting conclusions about ECMO as an independent risk factor.

Given associations between dnDSA and antibody mediated rejection (AMR) and chronic lung allograft dysfunction (CLAD), understanding dnDSA risk factors may impact screening protocols. However, even if ECMO is associated with dnDSA development, the clinical impact of these antibodies is unclear. Some exposures, such as influenza vaccination, do not induce clinically significant HLA antibodies, and not all dnDSAs carry the same clinical risk.^4^ There is a need to understand the relationship between ECMO bridge, subsequent humeral immune response, and adverse posttransplant outcomes, if any.

The primary objective of this study was to determine whether ECMO-bridged lung transplant recipients were more likely to develop dnDSA. The secondary objective was to evaluate the impact of ECMO-associated dnDSAs on 2 posttransplant outcomes: AMR and CLAD-free survival.

## Patients and Methods

### Cohort

This was a retrospective cohort study of all lung transplant recipients at the Hospital of the University of Pennsylvania from May 1, 2015 to June 30, 2021. Recipients were divided into 2 groups: ECMO-bridged and non-ECMO exposed. Multiorgan recipients and patients with intraoperative and/or postoperative ECMO without preoperative bridge were excluded. Screening and management protocols and ECMO cannulation configurations are available as Supplemental Material.

### Outcomes

The primary outcome was dnDSA development in lung transplant recipients with and without ECMO bridge. Additional outcomes included probable or definitive AMR in recipients with dnDSA with or without ECMO bridge, CLAD-free survival among ECMO-bridged recipients with and without dnDSA, and CLAD-free survival among recipients with dnDSA with and without ECMO bridge.

### Covariates

We collected data on factors associated with dnDSA development including age, sex, race/ethnicity, native lung disease, sensitization, cytomegalovirus mismatch, and transfusions. We defined “higher transfusion volume” as receipt of ≥4 total units of packed red blood cells, fresh frozen plasma, and/or platelets posttransplant (ie, greater than the median number of blood products per patient). For CLAD-free survival analysis, we included composite acute cellular rejection (ACR) score (ie, sum of A grades per biopsy divided by total number of biopsies) as a covariate given the association between ACR and CLAD.

### Statistical Analysis

We assessed differences between ECMO-bridged and non-ECMO exposed groups using Wilcoxon rank-sum test for continuous variables and Pearson’s χ^2^ test for categorical variables.

We used Cox proportional hazard models to compare patients who did and did not develop dnDSA. ECMO bridge was our primary predictor variable with age, sex, race/ethnicity, sensitization, and higher transfusion volume as covariates. We used a Fine-Gray competing risk analysis to assess dnDSA development between groups, with death treated as a competing risk. We examined Schoenfeld residuals to confirm the proportional hazard assumption.

To evaluate the clinical significance of ECMO-associated dnDSAs, we used Cox proportional hazard models to assess differences in CLAD-free survival between ECMO-bridged recipients with and without dnDSA, and between those who developed dnDSA with and without ECMO bridge, using age and composite ACR score as covariates. We used Fisher’s exact test to assess probable or definitive AMR development among those who developed dnDSA with or without ECMO bridge. Analyses were performed using Stata V.16.1 software (StataCorp).

## Results

### Cohort Characteristics

**Figure 1:**
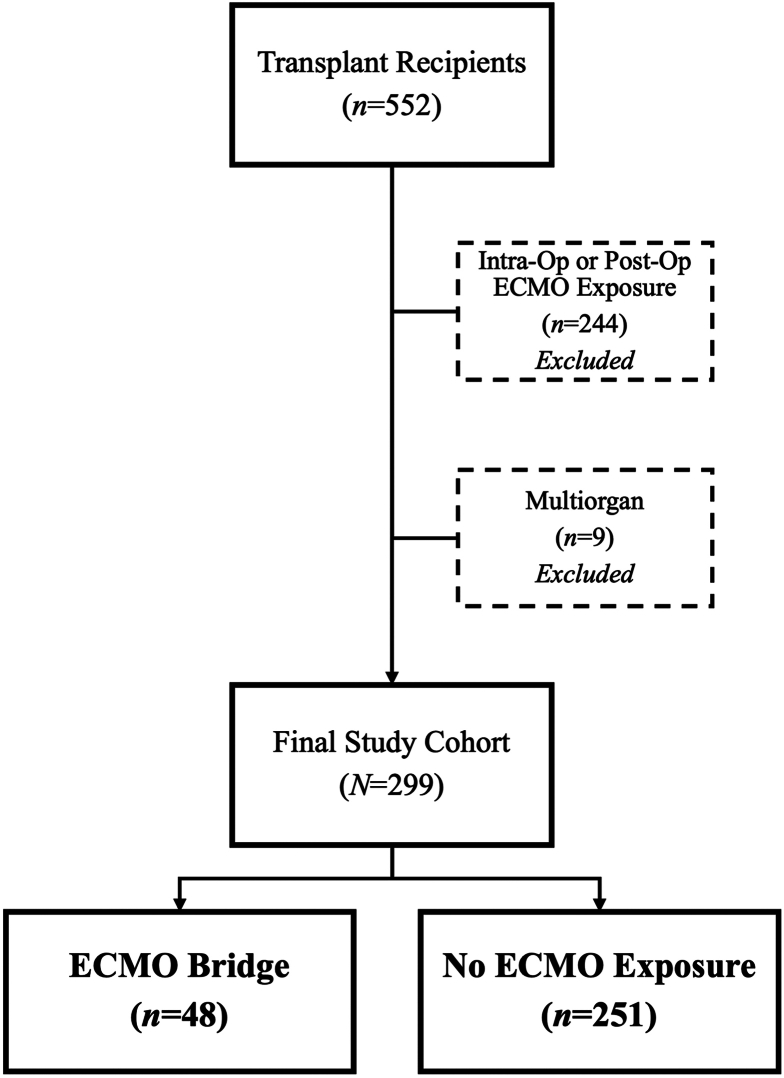
Study cohort. (ECMO, extracorporeal membrane oxygenation; Intra-Op, intraoperative; Post-Op, postoperative.)

There were 552 transplant recipients (Figure 1). Of those, 244 had intraoperative or postoperative ECMO exposure and were excluded. Nine multiorgan recipients were also excluded. The final cohort included 299 recipients, 48 (16.1%) of whom were ECMO-bridged. The remaining 251 non-ECMO exposed recipients served as the comparison cohort. The median follow-up time was 2.0 years (interquartile range [IQR], 0.9-3.9).

Sociodemographic and clinical characteristics are shown in Supplemental Table 1. ECMO-bridged recipients were significantly younger and more likely to be nonwhite and sensitized. Among ECMO-bridged recipients, 54.2% had higher posttransplant transfusion volume, compared with 7.2% of non-ECMO exposed recipients (*P* < .001). ECMO-bridged recipients received a median of 4 (IQR, 1.5-16) units of blood products, compared with 0 (IQR, 0-1) units in non-ECMO exposed recipients.

### dnDSA Development

Descriptive statistics summarizing dnDSA development, antibody class, and mean florescence intensity are shown in Supplemental Table 2. dnDSA developed in 33.3% of ECMO-bridged and 21.5% of non-ECMO exposed recipients, at a median of 60.5 (IQR, 15-89.5) days and 37 (IQR, 15-113) days (*P* = .94), respectively. Among ECMO-bridged recipients with dnDSA, 50.0% were treated, compared to 44.4% of non-ECMO exposed recipients with dnDSA (*P* = .70).

Results of Cox proportional hazard models assessing predictors of dnDSA development are shown in the Table. ECMO was associated with dnDSA development in bivariate (hazard ratio [HR], 2.08, 95% CI, 1.19-3.64, *P* = .01) but not multivariate analysis after adjusting for age, sex, race/ethnicity, sensitization, and higher transfusion volume (HR, 1.10, 95% CI, 0.50-2.42, *P* = .82). Higher transfusion volume was significantly independently associated with dnDSA (HR, 4.68, 95% CI, 2.25-9.72, *P* < .001). Additional analysis including pretransplant transfusion as a covariate showed no significant association (Supplemental Table 3). Fine-Gray subdistribution hazard models accounting for death as a competing risk for dnDSA development showed similar results (Supplemental Table 4). There was no significant difference in composite ACR score among ECMO-bridged and non-ECMO exposed recipients (median score, 0 [IQR, 0-0.35] vs 0.17 [IQR, 0-0.43], *P* = .44). Four (8.3%) ECMO-bridged recipients had probable or definitive AMR compared to 9 (3.6%) non-ECMO exposed recipients (*P* = .14). Among ECMO-bridged recipients with available composite ACR scores (n = 36), 10 experienced CLAD or died during the study period.

**Table:** Predictors of De Novo Donor-Specific Antibody Formation (N = 299)

Variable	Bivariate			Multivariate		
	HR	95% CI	*P* Value	HR	95% CI	*P* Value
Age	1.01	0.99-1.03	.22	1.02	1.00-1.05	.04
Female sex	1.14	0.71-1.85	.58	0.80	0.47-1.35	.40
Nonwhite	1.52	0.87-2.65	.15	1.12	0.61-2.07	.72
Sensitized	1.53	0.92-2.55	.10	1.44	0.86-2.40	.16
ECMO bridge	2.08	1.19-3.64	.01	1.10	0.50-2.42	.82
Higher posttransplant transfusion volume^a^	3.96	2.33-6.75	<.001	4.68	2.25-9.72	<.001
Days on ECMO pretransplant	0.98	0.96-1.01	.18	…	…	…

ECMO, extracorporeal membrane oxygenation; HR, hazard ratio.

a Defined as ≥4 total units of packed red blood cells, fresh frozen plasma, and/or platelets.

**Figure 2:**
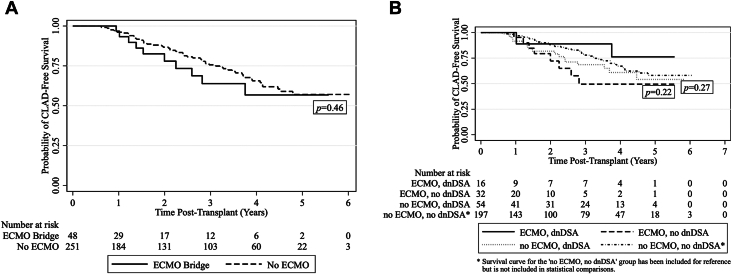
(A) Kaplan-Meier survival curves comparing chronic lung allograft dysfunction (CLAD)-free survival between extracorporeal membrane oxygenation (ECMO)-bridged and non-ECMO exposed recipients. (B) Kaplan-Meier survival curves comparing CLAD-free survival in ECMO-bridged recipients with and without de novo donor-specific HLA antibody (dnDSA) and non-ECMO exposed recipients who developed dnDSA.

ECMO-bridged recipients did not have worse CLAD-free survival than non-ECMO exposed recipients on unadjusted analysis (HR, 1.29, 95% CI, 0.66-2.52, *P* = .46) (Figure 2A). Among ECMO-bridged recipients with available composite ACR scores (n = 36), dnDSA development was not associated with worse CLAD-free survival on bivariate (HR, 0.32, 95% CI, 0.06-1.59, *P* = .16) or multivariate analysis controlling for age and composite ACR score (HR, 0.35, 95% CI, 0.07-1.87, *P* = .22). Among recipients with dnDSA with available composite ACR scores (n = 66), 18 experienced CLAD or died during the study period. In recipients with dnDSA, ECMO was not associated with worse CLAD-free survival on bivariate (HR, 0.47, 95% CI, 0.11-2.07, *P* = .32) or multivariate analysis controlling for age and composite ACR score (HR, 0.40, 95% CI, 0.08-2.00, *P* = .27). Kaplan-Meier survival curves comparing CLAD-free survival in ECMO-bridged recipients with and without dnDSA and non-ECMO exposed recipients with dnDSA are shown in Figure 2B.

## Comment

Despite the increasing use of ECMO bridge to lung transplant and evidence from the ventricular assist device literature suggesting mechanical circulatory support may cause immunologic priming, few studies have investigated whether ECMO impacts posttransplant humoral immunity. We found that ECMO-bridged lung transplant recipients were not more likely to develop dnDSA when considering other known risk factors. Further, ECMO-bridged recipients with dnDSA did not have worse CLAD-free survival than those without dnDSA or non-ECMO exposed recipients with dnDSA.

In our cohort, dnDSA rates (33.3% in ECMO-bridged, 21.5% in non-ECMO exposed) were consistent with other studies of lung transplant recipients, which have identified ranges from 12.2%-50.1% depending on screening protocols.^5^ dnDSA were detected at a median of 60.5 days for ECMO-bridged and 37 days for non-ECMO exposed recipients, which is consistent with prior research.^4^ Most identified dnDSAs were class II, and DQ was the most common specificity. This is consistent with prior research on dnDSA development in lung transplant recipients.^3^^,^^4^

Given the impact of dnDSA on AMR and CLAD, several cohort studies have identified risk factors for dnDSA formation.^3^^,^^4^ While we observed increased dnDSA risk among ECMO-bridged recipients, this association appeared to be moderated by higher posttransplant transfusion volumes. Prior studies investigating whether transfusions impact dnDSA development have found mixed results. For example, Islam and colleagues^6^ identified an association between postoperative platelet transfusion and dnDSA development, but did not adjust for potential confounders.

While we observed a strong relationship between postoperative transfusions and dnDSA development, there are several caveats. First, we have too few outcomes to assess whether specific blood products are higher risk, or whether the effect occurs beyond a specific transfusion threshold. Second, unmeasured variables may mediate the observed relationship. For example, recipients with postoperative bleeding often have other complications (eg, acute kidney injury) necessitating immunosuppression reduction, which may increase dnDSA risk. Third, while it is difficult to make uniform recommendations regarding transfusion goals based on these data alone, given multiple observations on the negative impacts of post-operative transfusions, a more conservative threshold may be appropriate.^7^

There is a growing literature on the clinical relevance of specific dnDSAs. For example, dnDSA targeted to the HLA-DQ antigen has been associated with adverse outcomes in solid organ transplant recipients, including CLAD development.^4^ We found that dnDSA among ECMO-bridged recipients did not decrease CLAD-free survival compared to those without dnDSA or non-ECMO exposed recipients with dnDSA. This effect persisted when considering death as a competing risk. Because we do not routinely treat dnDSA without allograft dysfunction and because rates of probable or definite AMR were similar between groups, the lack of relationship between ECMO-associated dnDSA and CLAD-free survival is not likely attributable to institution-specific management protocols.^8^ More plausibly, some dnDSAs self-resolve, target allograft antigens with low expressivity, do not fix complement, or are present in low titers. Understanding specific dnDSA behavior remains an underexplored area of lung transplantation research.

### Limitations

Our primary limitations were the cohort size and small number of outcomes. This study was thus only powered to detect large effect sizes. To minimize type I error, we performed a limited number of analyses. We were therefore unable to perform subgroup analyses, such as risk for dnDSA targeting certain HLA specificities. We were missing composite ACR scores for 23 recipients, which may have introduced bias into our CLAD-free survival analysis by disproportionately excluding sicker recipients less likely to survive to biopsy. Finally, our results are only generalizable to other centers using similar HLA antibody detection approaches.^5^

### Conclusions

A more restrictive posttransplant transfusion threshold among ECMO-bridged lung transplant recipients may reduce the risk of dnDSA. Surveillance for dnDSA, at least among ECMO-bridged recipients, may only be necessary in the presence of allograft dysfunction.
