# Supplementary material for: Donor Specific Antibodies in Extracorporeal Membrane Oxygenation-Bridged Lung Transplant Recipients
Source: Ann Thorac Surg Short Rep. 2024 Jul 4;2(4):836–41. doi: 10.1016/j.atssr.2024.06.021 (PMC11708649; doi:10.1016/j.atssr.2024.06.021)
Supplement: Supplementary Material [file mmc1.docx]

**Supplemental Material**

**Screening and Management Protocols**

All recipients underwent HLA antibody screening before and every 3 months following listing, with sensitized patients screened monthly. Samples were analyzed with the LABscreen Single Antigen assay (One Lambda, Canoga Park, California) without pre-screen. Antibodies present at mean fluorescence intensity (MFI) between 1000-3000 were considered moderate strength and to be avoided, if feasible. Antibodies present at MFI>3000 were considered unacceptable risk antibodies.

Extracorporeal membrane oxygenation (ECMO)-bridged recipients received HLA antibody screening weekly after ECMO initiation. Transfusions were avoided, where possible, with a target hemoglobin of 6 g/dL unless otherwise clinically indicated. All recipients received solumedrol and basiliximab as induction immunosuppression. The decision to cross moderate-risk DSA at the time of transplant was made on a case-by-case basis but did not alter initial immunosuppression management. All recipients underwent retrospective crossmatch within 48 hours post-transplant. Transfusions were administered according to clinical circumstances post-transplant without pre-specified targets.

Routine maintenance immunosuppression included steroids, a calcineurin inhibitor (most commonly tacrolimus), and a cell cycle inhibitor (most commonly mycophenolate mofetil). Recipients received DSA screening within two weeks post-transplant, at each surveillance bronchoscopy (typically at 3, 6, and 12 months), and with clinical changes in allograft function. Patients were considered to have dnDSA if donor-directed HLA antibodies were present at MFI>1000 post-transplant. dnDSA were not routinely treated with intravenous immunoglobulin (IVIG) unless there was concurrent hypogammaglobulinemia with ongoing infections. Recipients with dnDSA and evidence of allograft dysfunction consistent with probable or definitive AMR were treated with plasmapheresis, IVIG, and B cell depleting therapy.

**ECMO Cannulation Configurations**

Regarding ECMO support platforms, the majority of venovenous (VV) ECMO configurations were either standard internal jugular vein and femoral vein cannulation or internal jugular vein dual lumen cannulation (dlVV). During the COVID-19 pandemic, there was a shift toward dlVV ECMO with oxygenation-right ventricular assist device (oxy-RVAD) configurations because of secondary pulmonary hypertension. All venoarterial (VA) ECMO-bridged recipients underwent internal jugular and/or femoral vein cannulation combined with femoral artery cannulation.

**Supplemental Figure 1.** Fine-Gray subdistribution hazard model predicting de novo donor specific antibody (dnDSA) development with death treated as a competing risk. ECMO bridged candidates were not significantly more likely to develop dnDSA when controlling for age, gender, race/ethnicity, pre-transplant sensitization, and higher transfusion volume (sHR=1.17, 95% CI=0.58-2.34, *p*=0.66).

**Supplemental Table 1.** Sociodemographic and clinical characteristics of study cohorts (*N*=299)

|  | ECMO-Bridged  (*n*=48) | Non-ECMO Exposed  (*n*=251) | *p*-value |
| --- | --- | --- | --- |
| Age (years), median (IQR) | 50 (37-57) | 62 (55-67) | <0.001 |
| Female, *n* (%) | 19 (39.6) | 87 (34.7) | 0.51 |
| Nonwhite race/ethnicity, *n* (%) | 18 (37.5) | 36 (14.3) | <0.001 |
| Sensitized, *n* (%) | 17 (35.4) | 52 (20.7) | 0.03 |
| Native lung disease |  |  |  |
| Bronchiectasis, *n* (%) | 7 (14.6) | 30 (12.0) | <0.001 |
| Chronic obstructive pulmonary disease, *n* (%) | 0 (0.0) | 74 (29.5) |  |
| Interstitial lung disease, *n* (%) | 33 (68.8) | 129 (51.4) |  |
| Pulmonary hypertension, *n* (%) | 3 (6.3) | 4 (1.6) |  |
| Other, *n* (%) | 5 (10.4) | 14 (5.6) |  |
| CMV mismatch, *n* (%) | 9 (18.8) | 43 (17.1) | 0.79 |
| Time on ECMO pre-transplant (days), median (IQR) | 8 (4-19.5) | - | - |
| Time on ECMO total (days), median (IQR) | 10 (5.5-25) | - | - |
| ECMO bridge characteristics |  |  |  |
| Venovenous (VV), *n* (%) | 33 (68.8) | - | - |
| Venoarterial (VA), *n* (%) | 10 (20.8) | - | - |
| VA + VV, *n* (%) | 4 (8.3) | - | - |
| Extracorporeal carbon dioxide removal (ECCO2R), *n* (%) | 1 (2.1) | - | - |
| Retrospective T cell crossmatch positive, *n* (%) | 1 (2.1) | 8 (3.2) | - |
| Retrospective B cell crossmatch positive, *n* (%) | 2 (4.2) | 12 (4.8) | - |
| Higher post-transplant transfusion volume,* *n* (%) | 26 (54.2) | 18 (7.2) | <0.001 |

*Defined as ≥4 total units of packed red blood cells, fresh frozen plasma, and/or platelets.

CMV=cytomegalovirus; ECMO=extracorporeal membrane oxygenation; HLA=human leukocyte antigen; IQR=interquartile range

**Supplemental Table 2.** De novo donor-specific antibodies post-transplant, by study cohort (*N*=299)

|  | ECMO-Bridged  (*n*=48) | Non-ECMO Exposed  (*n*=251) |
| --- | --- | --- |
| Time to first DSA screen (days), median (IQR) | 8 (7-17) | 9 (7-19) |
| Number of DSA screens, median (IQR) | 5 (3-8) | 5 (3-7) |
| dnDSA Development, *n* (%) | 16 (33.3) | 54 (21.5) |
| Class I, *n* (%) | 7 (43.8) | 23 (42.6) |
| A | 3 | 3 |
| B | 4 | 16 |
| C | 3 | 9 |
| Class II, *n* (%) | 13 (81.3) | 40 (74.1) |
| DP | 3 | 3 |
| DQ | 13 | 38 |
| DR | 2 | 4 |
| Time to positive dnDSA (days), median (IQR) | 60.5 (15-89.5) | 37 (15-113) |
| MFI, mean (SD) | 5969 (5602) | 5295 (5734) |
| MFI, median (IQR) | 3200 (1400-9300) | 2800 (1700-6050) |
| MFI>10000, *n* (%) | 6 (37.5) | 8 (14.8) |

dnDSA=de novo donor-specific HLA antibody; ECMO=extracorporeal membrane oxygenation; HLA=human leukocyte antigen; MFI=mean fluorescence intensity; IQR=interquartile range; SD=standard deviation

**Supplemental Table 3.** Multivariable Cox proportional hazard model predicting de novo donor-specific antibody formation, including pre-transplant transfusion as a covariate (*N*=299)

| Variable | HR | 95% CI | *p*-value |
| --- | --- | --- | --- |
| Age | 1.02 | 1.00-1.05 | 0.04 |
| Female gender | 0.87 | 0.51-1.47 | 0.60 |
| Nonwhite | 1.16 | 0.63-2.13 | 0.64 |
| Sensitized | 1.53 | 0.91-2.56 | 0.11 |
| ECMO bridge | 1.56 | 0.64-3.84 | 0.33 |
| Higher post-transplant transfusion volume* | 4.61 | 2.26-9.42 | <0.001 |
| **Pre-transplant transfusion**** | **0.56** | **0.25-1.23** | **0.15** |

*Defined as ≥4 total units of packed red blood cells, fresh frozen plasma, and/or platelets.

**Defined as ≥1 unit of packed red blood cells, fresh frozen plasma, and/or platelets.

CI=confidence interval; ECMO=extracorporeal membrane oxygenation; HLA=human leukocyte antigen; HR=hazard ratio

**Supplemental Table 4.** Bivariate and multivariate Fine-Gray subdistribution hazard models for de novo donor specific antibody development with death treated as a competing risk (*N* = 299)

| Variable | Bivariate | | | Multivariate | | |
| --- | --- | --- | --- | --- | --- | --- |
|  | sHR | 95% CI | *p*-value | sHR | 95% CI | *p*-value |
| Age | 1.00 | 0.99-1.02 | 0.59 | 1.02 | 1.00-1.03 | 0.10 |
| Female gender | 1.32 | 0.82-2.13 | 0.25 | 1.21 | 0.76-1.94 | 0.42 |
| Nonwhite | 1.42 | 0.82-2.47 | 0.21 | 1.37 | 0.80-2.35 | 0.26 |
| Sensitized | 1.79 | 1.07-2.99 | 0.03 | 1.65 | 0.98-2.77 | 0.06 |
| ECMO bridge | 1.70 | 0.98-2.95 | 0.06 | 1.17 | 0.58-2.34 | 0.66 |
| Higher post-transplant transfusion volume* | 2.51 | 1.50-4.18 | <0.001 | 2.31 | 1.23-4.35 | 0.01 |

*Defined as ≥4 total units of PRBC, FFP, and/or platelets.

CI = confidence interval; ECMO = extracorporeal membrane oxygenation; FFP = fresh frozen plasma; HLA = human leukocyte antigen; PRBC = packed red blood cells; sHR = subhazard ratio
